# Factors Associated with Severe Leptospirosis, Martinique, 2010–2013

**DOI:** 10.3201/eid2112.141099

**Published:** 2015-12

**Authors:** Patrick Hochedez, Rafaelle Theodose, Claude Olive, Pascale Bourhy, Guillaume Hurtrel, Nicolas Vignier, Hossein Mehdaoui, Ruddy Valentino, Roland Martinez, Jean-Marie Delord, Cécile Herrmann, Isabelle Lamaury, Raymond Césaire, Mathieu Picardeau, André Cabié

**Affiliations:** Centre Hospitalier Universitaire de Martinique, Fort de France, Martinique, France (P. Hochedez, R. Theodose, C. Olive, G. Hurtrel, N. Vignier, H. Mehdaoui, R. Valentino, R. Césaire, A. Cabié);; Université des Antilles et de la Guyane, Fort de France, Martinique, and Pointe à Pitre, Guadeloupe, France (P. Hochedez, R. Theodose, C Olive, C. Herrmann, I. Lamaury, R. Césaire);; Institut Pasteur, Paris, France (P. Bourhy, M. Picardeau);; Centre Hospitalier Universitaire Avicenne, Bobigny, France (N. Vignier);; Centre Hospitalier de Trinité, Trinité, Martinique (R. Martinez);; Centre Hospitalier du Lamentin, Lamentin, Martinique (J.-M. Delord);; Centre Hospitalier Universitaire de Pointe à Pitre, Guadeloupe (C. Herrmann, I. Lamaury);; Institut National de la Santé et de la Recherche Medicale, Paris (A. Cabié)

**Keywords:** severe leptospirosis, leptospiremia, *Leptospira interrogans*, serogroup Icterohaemorrhagiae, quantitative PCR, qPCR, Martinique, bacteria

## Abstract

To identify factors associated with disease severity, we examined 102 patients with quantitative PCR–confirmed leptospirosis in Martinique during 2010–2013. Associated factors were hypotension, chest auscultation abnormalities, icterus, oligo/anuria, thrombocytopenia, prothrombin time <68%, high levels of leptospiremia, and infection with *L. interrogans* serovar Icterohaemorrhagiae/Copenhageni.

Leptospirosis is a bacterial zoonosis of worldwide distribution; incidence is highest in impoverished populations in developing countries and tropical regions (*1*). Humans are usually infected through contact with water or soil contaminated with the urine of carrier animals (*2*). The disease is caused by pathogenic strains of bacteria of the genus *Leptospira*, which is composed of 21 genomic species; 9 of them are pathogenic and comprise >200 serovars (*3*). To reduce the effects of severe leptospirosis, early diagnosis and prompt triage of high-risk patients is critical. Quantitative PCR (qPCR) might provide rapid diagnosis during the acute stage of the illness, offers the ability to measure the level of leptospiremia, and provides genomic identification (*4*–*6*). Our objectives were to determine if qPCR-determined leptospiremia was associated with severe evolution of the disease and to identify clinical and biological variables associated with severity.

## The Study

From December 2010 through February 2013, blood samples were obtained from a cohort of 102 adult patients with qPCR-confirmed leptospirosis at the University Hospital of Martinique. The study was approved by the French ethics committee. At the time of admission, clinical characteristics, biological findings, and potential exposures were recorded. Severe leptospirosis was defined by the presence of >1 of the following: shock treated with vasoactive drugs, acute renal failure requiring dialysis, internal bleeding requiring blood transfusion, respiratory insufficiency requiring mechanical ventilation, or death.

After EDTA-treated plasma was concentrated by centrifugation, DNA was extracted and used to perform a SYBR green assay (Bio-Rad, Hercules, CA, USA) selective for *lfb1* as previously described (*7*–*9*). The sensitivity of the assay was evaluated by using DNA extracted from 10-fold dilutions of reference strains (at 10^7^–10^2^ leptospires/mL) belonging to *L. borgpetersenii*, *L. interrogans*, and *L. kirschneri*. Serum samples were subjected to microscopic agglutination testing, and 45 available samples of *Leptospira* were cultured as previously described (*8*). Genomic DNA was extracted from cultures or from human plasma, and then *Leptospira* species and subspecies were identified as previously described (*10*,*11*).

Statistical analyses were performed by using Stata software version 12 (StataCorp LP, College Station, TX, USA). Leptospiremia was log-transformed. Receiver operating characteristics curve analysis was used to determine the critical threshold for leptospiremia as the marker for severity. Logistic regression was used to identify factors associated with severity. Continuous variables were summarized by using median, first quartile, and third quartile and compared by using nonparametric tests (Mann-Whitney or Kruskal-Wallis, as appropriate). A p value of <0.05 was considered statistically significant.

Most (86.3%) of the 102 patients were men; median age was 49 (37–57) years. Of these patients, 89 were hospitalized, 23 required treatment in intensive care units, and 12 (11.7%) had severe leptospirosis according to our clinical definition. The median delay between symptom onset and qPCR diagnosis was 3 days (first quartile and third quartile = 2, 5 days, respectively); blood tests were sampled from day 1 through day 11 after symptom onset, before administration of antimicrobial drugs. The median delay between symptom onset and antimicrobial drug receipt was 4 (3, 5) days. This delay did not differ significantly among patients with severe disease.

**Figure 1:**
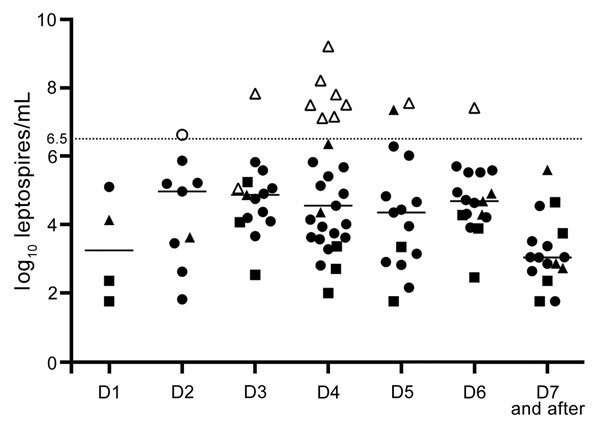
Leptospiremia in 102 patients with quantitative PCR–confirmed leptospirosis and day of sample collection since symptom onset, Martinique, 2010–2013. Each symbol (triangle, circle, or square) represents the leptospiremia of 1 leptospirosis patient on the day when the sample was collected. D indicates day since symptom onset. Open symbols indicate severe cases; closed symbols indicate nonsevere cases. Triangles correspond to *Leptospira interrogans* species, circles to other identified species, and squares to cases without genomic identification. Dotted line indicates the threshold for severe diseases determined by receiver operating characteristic curve analysis.

**Figure 2:**
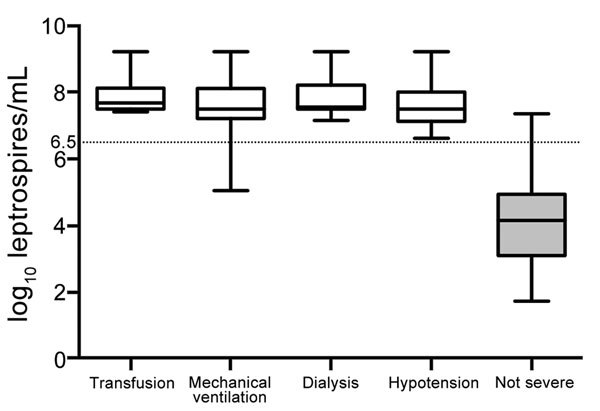
Distribution of leptospiremia among 102 patients with quantitative PCR–confirmed leptospirosis, grouped by severity criteria, Martinique, 2010–2013. Criteria that met our clinical definition for severe leptospirosis were shock treated with vasoactive drugs, acute renal failure requiring dialysis, internal bleeding requiring blood transfusion (e.g., alveolar hemorrhage), and respiratory insufficiency requiring mechanical ventilation or death during hospitalization. Horizontal lines in box-and-whisker plots indicate (top to bottom) maximum value, third quartile, median (second quartile), first quartile, minimum value. Dotted line indicates the threshold for severe diseases determined by receiver operating characteristic curve analysis.

Leptospiremia, determined by qPCR (Figure 1), was significantly higher among patients with severe disease (7.49 log_10_ [7.13, 7.81] vs. 4.16 log_10_ [3.14, 4.93]; p = 0.00001). Among those with severe disease, 9 had shock requiring vasoactive drugs, 8 had pulmonary involvement requiring mechanical ventilation, 8 had internal bleeding requiring blood transfusion, and 7 had acute renal failure requiring dialysis. No patient died. The median length of evolution before occurrence of severe leptospirosis was 3 (3, 4) days. Using a receiver operating characteristic curve analysis, we found a critical threshold of 6.5 log_10_ leptospires/mL that could be considered severe leptospirosis (Figures 1, 2). Except for acute renal failure, all complications were associated with a higher level of leptospiremia (Technical Appendix Table 1).

The only epidemiologic characteristic associated with severity was presence of rats in the house or the surrounding vicinity (p = 0.02). Clinical and biological findings recorded at admission were associated with severity (Tables 1, 2) as follows: hypotension, chest auscultation abnormalities, icterus, oligo/anuria, bilirubin >49 μmol/L, creatinine >154 μmol/L, creatine phosphokinase >443 U/L, C-reactive protein >282 mg/L, hemoglobin <12.2 g/dL, lymphocytes <0.49 × 10^9^ cells/L, platelets <92 × 10^9^/L, and prothrombin time <68%.

**Table 1:** Clinical characteristics of 102 patients with quantitative PCR–confirmed leptospirosis, by disease severity, Martinique, 2010–2013

Characteristic	All patients, n = 102, no. (%)	Patients with severe disease, n = 12, no. (%)	Patients with nonsevere disease, n = 90, no. (%)	p value
Fever >38°C	88 (86.3)	9 (75)	79 (87.8)	0.364
Hypotension*	10 (9.8)	5 (41.7)	5 (5.6)	0.002
Cough	12 (11.8)	3 (25)	9 (10)	0.148
Abnormalities at chest auscultation	7 (6.9)	4 (33.3)	3 (3.3)	0.003
Abdominal pain	30 (29.4)	5 (41.7)	25 (27.8)	0.329
Vomiting	42 (41.2)	5 (41.7)	37 (41.1)	1
Diarrhea	30 (29.4)	3 (25)	27 (30)	1
Icterus	39 (38.2)	9 (75)	30 (33.3)	0.009
Conjunctival suffusion	20 (19.6)	1 (8.3)	19 (21.1)	0.45
Consciousness disorders	2 (1.6)	1 (8.3)	1 (1.1)	0.2
Hemorrhage†	6 (5.9)	1 (8.3)	5 (5.6)	0.54
Oliguria** or anuria‡	8 (7.8)	5 (41.7)	3 (3.3)	0.0001

*Systolic blood pressure <90 mm Hg. †Hemoptysis, hematuria, bleeding of the gums, or hematemesis. ‡<500 mL urine/day.

**Table 2:** Initial laboratory findings among 102 patients with quantitative PCR–confirmed leptospirosis, by disease severity, Martinique, 2010–2013

Initial laboratory findings*	All patients, n = 102, no. (%)	Patients with severe disease, n = 12, no. (%)	Patients with nonsevere disease, n = 90, no. (%)	p value
Bilirubin				
μmol/L (Q1, Q3)	20 (12, 49)	56.5 (35.5, 103)	18 (12, 38)	0.0035
>49 μmol/L, no./total (%)	25/99 (25.2)	7/12 (58.3)	18/87 (20.7)	0.01
Creatinine				
μmol/L (Q1, Q3)	104 (88, 154)	169.5 (132.5, 217.5)	100 (87, 137)	0.0084
>154 μmol/L, no./total (%)	26/101 (25.7)	7/12 (58.3)	19/89 (21.3)	0.011
Urea nitrogen (mmo/Ll)				
mmol/L (Q1, Q3)	5.7 (4.2, 9.3)	10.1 (8, 18.5)	5.5 (4, 8.6)	0.0068
>9.3, mmol/L, no./total (%)	21/84 (25)	4/8 (50)	17/76 (22.4)	0.103
Creatine phosphokinase				
U/L (Q1, Q3)	170 (70, 443)	953 (204, 1332)	145 (64, 390)	0.0202
>443 U/L, no./total (%)	19/75 (25.3)	5/9 (55.6)	14/66 (21.2)	0.041
C-reactive protein				
mg/L (Q1, Q3)	188.5 (108, 282)	338.5 (197.5, 464.5)	177.9 (89, 265)	0.0017
>282 mg/L, no./total (%)	26/102 (25.5)	7/12 (58.3)	19/90 (21.1)	0.011
Potassium, mmol/ L (Q1, Q3)	3.7 (3.4, 4.1)	3.75 (3.35, 4.15)	3.7 (3.3, 4.1)	0.8
Sodium, mmo/L (Q1, Q3)	134 (132, 136)	134 (131.5, 135)	134 (132, 136)	0.44
Aspartate aminotransferase, U/L (Q1, Q3)	61.5 (32, 102)	73.5 (59, 126.5)	57.5 (31, 102)	0.19
Alanine aminotransferase, U/L(Q1, Q3)	55 (30, 96)	49 (33.5, 74.5)	55 (30, 99)	0.69
Hemoglobin				
g/dL (Q1, Q3)	13.2 (12.2, 14.5)	12.2 (11.6, 13)	13.3 (12.4, 14.7)	0.027
<12.2 g/dL, no./total (%)	26/102 (25.5)	6/12 (50)	20/90 (22.2)	0.071
Leukocytes, × 10^9^ cells/L (Q1, Q3)	8.51 (6.2, 10.9)	10.3 (9.1, 11.4)	7.8 (6.1, 10.5)	0.07
Lymphocytes				
× 10^9^ cells/L (Q1, Q3)	0.7 (0.49, 1)	0.5 (0.2, 0.7)	0.7 (0.5, 1)	0.043
<0.49 × 10^9^ cells/L, no./total (%)	24/92 (26)	4/8 (50)	20/84 (23.8)	0.19
Platelets				
Concentration, × 10^9^/L (Q1, Q3)	138 (92, 183)	70.5 (32.5, 115)	141 (99, 191)	0.0011
<92 × 10^9^/L, no./total (%)	26/101 (25.7)	7/12 (58.3)	19/89 (21.3)	0.011
Prothrombin time				
% (Q1, Q3)	74 (68, 90.5)	66.5 (56, 74.5)	75.5 (69, 91)	0.0166
<68%, no./total (%)	20/76 (26.3)	7/12 (58.3)	13/64 (20.3)	0.011

*Continuous variables are summarized by using median, first quartile (Q1), and third quartile (Q3). Hematologic and biochemical variables are categorized into 2 groups, using Q1 or Q3 as appropriate.

Molecular typing of genomic DNA was performed from the 102 acute-phase blood samples (Technical Appendix Table 2). Leptospire species determination was successful for 85 (83%) patients and corresponded to 1 of the following 6 pathogenic species: *L. interrogans* (n = 23), *L. santarosai* (n = 22), *L. borgpetersenii* (n = 18), *L. kirschneri* (n = 15), *L. kmetyi* (n = 4), and *L. noguchii* (n = 3). Among the genomic species identified, *L. interrogans* was associated with severity (p = 0.001), highest level of leptospiremia (p = 0.0001), and previous exposure to rats (p = 0.02). The level of leptospiremia in specimens for which species was not identified was significantly lower (p = 0.0001). The median melting peak for *L. interrogans* strains was 83.1°C (82.8°C, 83.4°C), which differed significantly from that of other species, for which the median melting peak was 85°C (84°C, 85.9°C) (p = 0.0001).

Microscopic agglutination testing enabled identification of the putative serogroups (highest titer >400) for 70 (68.6%) patients; the 3 most frequently identified serogroups were Icterohaemorrhagiae (n = 39), Ballum (n = 11), and Celledoni (n = 10). Serogroup Icterohaemorrhagiae can be subdivided into serovars Icterohaemorrhagiae/Copenhageni (n = 20) and Bogvere (n = 10); the remaining 9 serogroups cannot be unambiguously typed at the serovar level. Serovar Icterohaemorrhagiae/Copenhageni was identified for 11 of the 12 patients with severe disease (p = 0.03). The identification of the putative serogroup was not possible for 32 patients (Technical Appendix Table 3).

## Conclusions

This prospective study enabled us to report the potential contribution of qPCR to timely diagnosis and leptospirosis severity evaluation at the point of care in a disease-endemic area. We based our classification of severity on treatment-related criteria to reflect everyday patient management, as previously reported (*12*,*13*). The fact that no patient died could be associated with factors such as reduced diagnosis time and early treatment. Currently, only qPCR enables unequivocal diagnosis during the acute phase of illness, when antimicrobial drugs are most likely to have the greatest benefit (*6*,*14*). Our results show a strong association between leptospiremia levels and disease severity. A lower critical threshold was reported in New Caledonia, and differences between critical thresholds may be associated with the variability of virulence among serovars, host factors, or qPCR technique (*13*).

The samples used for qPCR diagnosis were also used for direct *Leptospira* genomic identification, although molecular typing performance was impaired for samples with the lowest leptospiremia, as previously reported (*15*). The factors significantly associated with severity were infection with the species *L. interrogans,* the serogroup Icterohaemorrhagiae, and the presence of rats (usual carriers of that serogroup). In that context, melting curve analysis of the assay may provide rapid and useful additional information because it can differentiate between *L. interrogans* and other pathogenic species (*7*,*9*). The potential correlation between disease severity and serogroup Icterohaemorrhagiae has been reported in other tropical islands, and our results also emphasize the need for public health action to control rodents (*12*,*13*).

qPCR can be used for rapid diagnosis of acute leptospirosis and may provide timely information useful for evaluation of disease severity. Use of qPCR to determine leptospiremia seems increasingly accessible and should be evaluated in other disease-endemic areas. Whether high levels of leptospiremia are associated with factors such as pathogen virulence characteristics or host factors should also be explored.

Technical Appendix Complications in 102 leptospirosis patients and associated leptospiremia; genomic identification based on 16S rRNA sequencing of PCR products and the relative leptospiremia; and serogroup and genomic identification in 102 patients with quantitative PCR–confirmed leptospirosis, Martinique, 2010–2013.
