# Supplementary material for: Factors Associated with Severe Leptospirosis, Martinique, 2010–2013
Source: Emerg Infect Dis. 2015 Dec;21(12):2221–4. doi: 10.3201/eid2112.141099 (PMC4672444; doi:10.3201/eid2112.141099)
Supplement: Technical Appendix — Complications in 102 leptospirosis patients and associated leptospiremia; genomic identification based on 16S rRNA sequencing of PCR products and the relative leptospiremia; and serogroup and genomic identification in 102 patients with quantitative PCR–confirmed leptospirosis, Martinique, 2010–2013. [file 14-1099-Techapp-s1.pdf]

Article DOI: http://dx.doi.org/10.3201/eid2112.141099

Severe Leptospirosis in Martinique, 2010–2013

Technical Appendix

Technical Appendix Table 1. Complications in 102 confirmed cases of leptospirosis and their associated leptospiremia in Martinique, 2010-2013

Complications	Cases, n (%); Leptospiremia, log_{10}/ml	Controls, n (%); Leptospiremia, log_{10}/ml	P value
Icteric leptospirosis	40 (39%)<br>4.88 (3.91-6.86)	62 (61%)<br>4.13 (3.14-4.90)	0.0035
Acute renal failure	28 (27%)<br>4.77 (3.55-7.28)	74 (73%)<br>4.25 (3.34-5.09)	0.09
Multi organ failure	24 (23%)<br>5.55 (4.07-7.49)	78 (77%)<br>4.16 (3.34-4.96)	0.0052
Arterial hypotension	22 (21%)<br>6.10 (4.55-7.50)	80 (79%)<br>4.13 (3.20-4.93)	0.0002
Internal haemorrhage	11 (11%)<br>7.50 (3.88-7.82)	91 (89%)<br>4.29 (3.34-5.09)	0.0023
Shock treated with vasoactive drugs *	9 (9%)<br>7.49 (7.15-7.80)	93 (91%)<br>4.21 (3.27-5.05)	<0.0001
Respiratory insufficiency requiring mechanical ventilation*	8 (8%)<br>7.49 (7.28-8)	94 (92%)<br>4.24 (3.27-5.09)	<0.0001
Alveolar haemorrhage	8 (8%)<br>7.53 (7.45-8.01)	94 (92%)<br>4.28 (3.27-5.09)	0.0001
Internal bleeding requiring blood transfusion*	8 (8%)<br>7.68 (7.5-8.02)	94 (92%)<br>4.24 (3.27-5.05)	<0.0001
Acute renal failure requiring dialysis*	7 (7%)<br>7.56 (7.49-8.21)	95 (93%)<br>4.27 (3.27-5.09)	<0.0001
Altered mental status	4 (4%)<br>7.49 (7.06-7.86)	98 (96%)<br>4.29 (3.34-5.19)	0.002

*Complications that met our clinical definition for severe leptospirosis.

Technical Appendix Table 2. Genomic identification based on 16S rRNA sequencing of PCR products, and the relative leptospiremia in confirmed cases of leptospirosis in Martinique, 2010-2013

Species	All cases, N=102	Cases with severe disease, N=12, no (%)	Leptospiremia, log_{10}/ml, median (p25-p75)
*L interrogans**	23	11 (48)	6.35 (4.35-7.50)
L santarosai	22	0	4.39 (3.62-4.94)
L borgpetersenii	18	1(6)	4.11 (2.85-5.21)
L. kirschneri	15	0	4.81 (3.90-5.58)
L. kmetyi	4	0	3.26 (2.41-4.02)
L. noguchii	3	0	4.21 (3.27-5.13)
Unidentified**	17	0	2.69 (2.34-3.88)

*Compared with other species, *L. interrogans* was associated with highest level of leptospiremia (P=0.0001).
** Leptospiremia of specimens without species identification was significantly lower (P=0.0001).

Technical Appendix Table 3. Serogroup and genomic identification in confirmed 102 cases of leptospirosis in Martinique, 2010-2013

Severity	Presumptive serogroup (serum) *[a]*	blood culture *[b]*	Serogroup (culture) *[c]*	blood DNA *[b]*	species (rrs)	genotype *[d]*	MLVA *[e]*	Presumptive serogroup/serovar *[f]*
1	Icterohaemorrhagiae			+	*L. interrogans*	no sequence	500/350/750	Ictero / Ictero
1	Icterohaemorrhagiae			+	*L. interrogans*	A	500/350/750	Ictero / Ictero
1	Icterohaemorrhagiae			+	*L. interrogans*	A	500/350/750	Ictero / Ictero
1	unknown (titer<400)			+	*L. interrogans*	A	500/350/750	Ictero / Ictero
1	Icterohaemorrhagiae			+	*L. interrogans*	A	500/350/750	Ictero / Ictero
1	ND			+	*L. interrogans*	A	500/350/750	Ictero / Ictero
1	Icterohaemorrhagiae	+	Ictero	+	*L. interrogans*	A	500/350/750	Ictero / Ictero
1	unknown (co-agglutination)			+	*L. interrogans*	A	500/350/750	Ictero / Ictero
1	Icterohaemorrhagiae			+	*L. interrogans*	A	500/350/750	Ictero / Ictero
1	Icterohaemorrhagiae	+	Ictero	+	*L. interrogans*	A	500/350/750	Ictero / Ictero
1	Icterohaemorrhagiae			+	*L. interrogans*	A	ND	Ictero / Ictero
1	Tarassovi	+	Tarassovi	+	*L. borgpetersenii*	no sequence	ND	Tarassovi / unknown
0	unknown (co-agglutination)			+	*L. kirschneri*	no sequence	ND	unknown / unknown
0	Icterohaemorrhagiae			+	*L. kirschneri*	no sequence	ND	Ictero / Bogvere
0	Ballum			+	*L. borgpetersenii*	no sequence	ND	Ballum / Arborea
0	Icterohaemorrhagiae			+	*L. kirschneri*	no sequence	ND	Ictero / Bogvere
0	Icterohaemorrhagiae	+	Celledoni	+	*L. santarosai*	I	ND	Celledoni / unknown
0	unknown (titer<400)	+	Tarassovi	+	*L. santarosai*	L	ND	Tarassovi / unknown
0	Ballum			+	*L. borgpetersenii*	no sequence	ND	Ballum / Arborea
0	Icterohaemorrhagiae			+	*L. borgpetersenii*	no sequence	ND	unknown / unknown
0	Celledoni			+	no sequence	no sequence	ND	Celledoni / unknown
0	unknown (titer<400)			+	*L. santarosai*	no sequence	ND	unknown / unknown
0	unknown (titer<400)			+	*L. santarosai*	no sequence	ND	unknown / unknown
0	Celledoni	+	unknown	+	*L. santarosai*	H	ND	unknown / unknown
0	Icterohaemorrhagiae			+	no sequence	no sequence	ND	Ictero / unknown
0	Icterohaemorrhagiae	+	Ictero	+	*L. interrogans*	A	500/350/750	Ictero / Ictero
0	Grippotyphosa			+	*L. interrogans*	no sequence	ND	unknown / unknown
0	Icterohaemorrhagiae			+	*L. interrogans*	A	500/350/750	Ictero / Ictero
0	ND			+	*L. kmetyi*	M	ND	unknown / unknown
0	ND			+	no sequence	no sequence	ND	unknown / unknown
0	unknown (titer<400)			+	*L. kmetyi*	no sequence	ND	unknown / unknown
0	Icterohaemorrhagiae			+	*L. borgpetersenii*	C	ND	Ballum / Arborea
0	unknown (titer<400)			+	no sequence	no sequence	ND	unknown / unknown

Severity	Presumptive serogroup (serum) *[a]*	blood culture *[b]*	Serogroup (culture) *[c]*	blood DNA *[b]*	species (rrs)	genotype *[d]*	MLVA *[e]*	Presumptive serogroup/serovar *[f]*
0	unknown (titer<400)			+	*L. noguchii*	E	ND	Australis / Bajan
0	unknown (co-agglutination)			+	*L. interrogans*	no sequence	ND	unknown / unknown
0	unknown (titer<400)			+	*L. borgpetersenii*	C	ND	Ballum / Arborea
0	unknown (co-agglutination)			+	*L. kirschneri*	B	380/560/0	Ictero / Bogvere
0	unknown (titer<400)			+	no sequence	no sequence	ND	unknown / unknown
0	unknown (titer<400)			+	*L. santarosai*	no sequence	ND	unknown / unknown
0	Ballum			+	*L. borgpetersenii*	C	ND	Ballum / Arborea
0	unknown (titer<400)			+	*L. kmetyi*	no sequence	ND	unknown / unknown
0	unknown (titer<400)			+	*L. santarosai*	I	ND	Celledoni / unknown
0	Icterohaemorrhagiae	+	Ballum	+	*L. borgpetersenii*	C	ND	Ballum / Arborea
0	Icterohaemorrhagiae	+	Ictero	+	*L. kirschneri*	B	380/560/0	Ictero / Bogvere
0	unknown (co-agglutination)			+	*L. borgpetersenii*	C	ND	Ballum / Arborea
0	Celledoni			+	*L. santarosai*	I	ND	Celledoni / unknown
0	Cynopteri			+	*L. borgpetersenii*	D	ND	Tarassovi / unknown
0	Icterohaemorrhagiae	+	Ictero	+	*L. interrogans*	A	500/350/750	Ictero / Ictero
0	unknown (titer<400)			+	*L. kirschneri*	no sequence	ND	unknown / unknown
0	unknown (titer<400)	+	Mini	+	*L. santarosai*	J	ND	unknown / unknown
0	Tarassovi			+	*L. santarosai*	no sequence	ND	unknown / unknown
0	unknown (titer<400)			+	*L. santarosai*	H	ND	unknown / unknown
0	unknown (titer<400)			+	*L. borgpetersenii*	C	ND	Ballum / Arborea
0	unknown (titer<400)			+	*L. kirschneri*	no sequence	ND	unknown / unknown
0	unknown (co-agglutination)			+	*L. interrogans*	no sequence	ND	unknown / unknown
0	unknown (titer<400)			+	*L. santarosai*	no sequence	ND	unknown / unknown
0	Sejroe			+	no sequence	no sequence	ND	unknown / unknown
0	Icterohaemorrhagiae			+	no sequence	no sequence	ND	Ictero / unknown
0	Icterohaemorrhagiae	+	Ictero	+	*L. kirschneri*	B	380/560/0	Ictero / Bogvere
0	Icterohaemorrhagiae			+	*L. kirschneri*	no sequence	ND	Ictero / Bogvere
0	Icterohaemorrhagiae			+	*L. kirschneri*	B	380/560/0	Ictero / Bogvere
0	unknown (titer<400)			+	*L. santarosai*	I	ND	Celledoni / unknown
0	unknown (titer<400)	+	Ictero	+	*L. interrogans*	A	500/350/750	Ictero / Ictero
0	Icterohaemorrhagiae			+	*L. santarosai*	J	ND	unknown / unknown
0	Celledoni			+	*L. santarosai*	I	ND	Celledoni / unknown
0	unknown (titer<400)	+	unknown	+	*L. santarosai*	J	ND	unknown / unknown
0	unknown (titer<400)			+	*L. santarosai*	H	ND	unknown / unknown
0	unknown (titer<400)	+	unknown	+	*L. santarosai*	H	ND	unknown / unknown
0	Tarassovi			+	*L. borgpetersenii*	D	ND	Tarassovi / unknown
0	Australis			+	*L. noguchii*	no sequence	ND	Australis / Bajan
0	Tarassovi			+	*L. borgpetersenii*	no sequence	ND	Tarassovi / unknown
0	Icterohaemorrhagiae	+	Ictero	+	*L. interrogans*	A	500/350/750	Ictero / Ictero
0	Hebdomadis	+	Tarassovi	+	*L. borgpetersenii*	D	ND	Tarassovi / unknown
0	Icterohaemorrhagiae			+	no sequence	no sequence	ND	Ictero / unknown
0	unknown (titer<400)			+	no sequence	no sequence	ND	unknown / unknown
0	Sarmin			+	*L. kirschneri*	no sequence	ND	unknown / unknown
0	Icterohaemorrhagiae			+	no sequence	no sequence	ND	Ictero / unknown
0	Ballum	+	Celledoni	+	*L. santarosai*	I	ND	Celledoni / unknown
0	Icterohaemorrhagiae			+	no sequence	no sequence	ND	Ictero / unknown
0	Icterohaemorrhagiae			+	no sequence	no sequence	ND	Ictero / unknown

Severity	Presumptive serogroup (serum) *[a]*	blood culture *[b]*	Serogroup (culture) *[c]*	blood DNA *[b]*	species (rrs)	genotype *[d]*	MLVA *[e]*	Presumptive serogroup/serovar *[f]*
0	unknown (co-agglutination)	+	Celledoni	+	*L. santarosai*	I	ND	Celledoni / unknown
0	unknown (co-agglutination)			+	*L. interrogans*	no sequence	500/350/750	Ictero / Ictero
0	unknown (negative)			+	*L. kmetyi*	no sequence	ND	unknown / unknown
0	Louisiana			+	*L. kirschneri*	no sequence	ND	unknown / unknown
0	Icterohaemorrhagiae			+	*L. kirschneri*	no sequence	ND	Ictero / Bogvere
0	Icterohaemorrhagiae			+	*L. borgpetersenii*	D	ND	Tarassovi / unknown
0	unknown (co-agglutination)	+	Ictero	+	*L. kirschneri*	no sequence	ND	Ictero / Bogvere
0	unknown (co-agglutination)			+	*L. noguchii*	E	ND	Australis / Bajan
0	Cynopteri			+	*L. interrogans*	A	500/350/750	Ictero / Ictero
0	unknown (titer<400)			+	*L. borgpetersenii*	C	ND	Ballum / Arborea
0	Canicola			+	no sequence	no sequence	ND	unknown / unknown
0	Icterohaemorrhagiae			+	no sequence	no sequence	ND	Ictero / unknown
0	Louisiana	+	Celledoni	+	*L. santarosai*	I	ND	Celledoni / unknown
0	Canicola			+	*L. borgpetersenii*	C	ND	Ballum / Arborea
0	Ballum			+	*L. borgpetersenii*	C	ND	Ballum / Arborea
0	Icterohaemorrhagiae			+	no sequence	no sequence	ND	Ictero / unknown
0	unknown (co-agglutination)	+	Celledoni	+	*L. santarosai*	I	ND	Celledoni / unknown
0	Icterohaemorrhagiae			+	*L. interrogans*	A	ND	Ictero / Ictero
0	unknown (negative)			+	no sequence	no sequence	ND	unknown / unknown
0	Icterohaemorrhagiae			+	no sequence	no sequence	ND	Ictero / unknown
0	unknown (co-agglutination)			+	*L. interrogans*	A	500/350/750	Ictero / Ictero
0	Icterohaemorrhagiae			+	*L. kirschneri*	no sequence	ND	Ictero / Bogvere

a: Serogroup was determined by MAT on serum samples (two sampling made >1week apart). High titers (>=400) of the serum with one particular antigen were used to identify the presumptive serogroup of the infecting bacterium.

b: PCR- and culture positive samples

c: Serogrouping of isolates by MAT with rabbit antisera against reference serovars of the main serogroups (Australis, Autumnalis, Bataviae, Canicola, Ballum, Cynopteri, Grippotyphosa, Sejroe, Hebdomadis, Icterohaemorrhagiae, Panama, Semaranga, Pomona, Pyrogenes, Tarassovi, Celledoni, Djamisan, Mini, Sarmin, Shermani, Javanica, and Louisiana)

d: genotype determined by sequencing of secY (see Bourhy et al. 2013)

e: size in bp of the PCR products for VNTR4, VNTR7, and VNTR10 (see Salaun et al. 2006)

f : When culture isolates are availables, we can perform serogrouping and molecular typing, including PFGE. In most cases, the identification of the serogroup and the PFGE profile enables the identification at the serovar level, except when no agglutination was obtained (the serogroup cannot be determined) or when the PFGE profile does not correspond to any reference serovar. When isolates are not availables, the serovar can be determined by molecular typing methods on biological samples: an identical secY sequence and MLVA profile with a known Leptospira serovar circulating in the Caribbean islands enable the identification of the presumptive serovar directly from the biological sample.

Ictero: Icterohaemorhagiae; for the serovar, Ictero can correspond to either serovar Icterohaemorhagiae or Copenhageni

ND: Not Done.
